# Historical and Philosophical Perspectives on Contemporary Biology

**DOI:** 10.1371/journal.pbio.0060321

**Published:** 2008-12-23

**Authors:** Evelyn Fox Keller

## Abstract

Evelyn Fox Keller introduces a new series that aims to promote productive dialogue between laboratory researchers and historians and philosophers of science to address the challenges arising from the rapid pace of biological discovery.

The work of historians and philosophers of science has long benefited from conversations with practicing scientists, but to many scientific researchers—perhaps especially to those engaged in laboratory work—the value that such dialogue might have for their own endeavor is not nearly so obvious. There are of course exceptions—evolutionary biology, for one. Over the last several decades, a tradition of active engagement between historians and philosophers on the one hand, and evolutionary biologists on the other, has become well established, and, as most participants would surely agree, this tradition has proven to be of manifest and clearly mutual benefit. The new series, Historical and Philosophical Perspectives, launched in this issue of *PLoS Biology*, provides the opportunity to help promote a similar engagement in other areas of biology. This opportunity is both welcome and timely: not only do I believe it is long overdue (there is, after all, no reason to regard evolutionary biology as a special case in this regard), but also, such an opportunity seems to me to be especially propitious today.

It has been said that biologists are used to surprises in their research, but rarely has the pace at which startling, even earthshaking, new discoveries are reported equaled that which we have seen in the biological literature over the last twenty years. Many of these new findings upset some of our oldest and most cherished assumptions about the nature of biological processes, causing us to rethink accepted theories of heredity, of development, and even of evolution. At such a time, it can often be useful to cast a wide eye—both backward over the past history of biological thought, and sideways, over the kinds of analyses that philosophers of biology have to offer.

There is also another reason for the timeliness of this series, and it is of an altogether different kind: never before has the impact of developments in the biological sciences on our social and economic life been so rapid or so dramatic. Again, a historical perspective may provide us with useful insights, as might the perspective offered by social scientists trained in the study of social and economic change.

The aim of the series launched in this issue of *PLoS Biology* is to encourage productive dialogue between laboratory researchers and historians and philosophers about the many challenges before us. Our hope is that, through such dialogue, we can all broaden our horizons in ways that will facilitate the meeting of these challenges. In the first essay, published today (doi:10.1371/journal.pbio.0060320), Diane Paul (University of Massachusetts, Boston, United States) and Hamish Spencer (University of Otago, New Zealand) provide a historical perspective on conflicts over consanguinity. Here they explore the history of scientific and public opinion on human inbreeding in the United States where, uniquely in the Western world, 32 states currently prohibit or restrict the marriage of first cousins. Contributions to the series from other authors are encouraged; ideas should be sent to biology_editors@plos.org.

